# Association of Breast Density With Breast Cancer Risk Among Women Aged 65 Years or Older by Age Group and Body Mass Index

**DOI:** 10.1001/jamanetworkopen.2021.22810

**Published:** 2021-08-26

**Authors:** Shailesh M. Advani, Weiwei Zhu, Joshua Demb, Brian L. Sprague, Tracy Onega, Louise M. Henderson, Diana S. M. Buist, Dongyu Zhang, John T. Schousboe, Louise C. Walter, Karla Kerlikowske, Diana L. Miglioretti, Dejana Braithwaite

**Affiliations:** 1 Department of Oncology, Georgetown University, Washington, DC; 2 Terasaki Institute of Biomedical Innovation, Los Angeles, California; 3 Kaiser Permanente Washington Health Research Institute, Seattle, Washington; 4 Department of Medicine, University of California, San Diego; 5 Department of Surgery, Larner College of Medicine, University of Vermont, Burlington; 6 Department of Population Sciences, University of Utah, Salt Lake City; 7 Department of Radiology, University of North Carolina at Chapel Hill; 8 Department of Epidemiology, University of Florida, Gainesville; 9 Division of Research, Health Partners Institute, Bloomington, Minnesota; 10 Department of Medicine, University of California, San Francisco; 11 Department of Epidemiology and Biostatistics, University of California, San Francisco; 12 Department of Public Health Sciences, School of Medicine, University of California, Davis; 13 Cancer Control and Population Sciences Program, University of Florida Health Cancer Center, Gainesville; 14 Department of Epidemiology, University of Florida, Gainesville

## Abstract

**Importance:**

Breast density is associated with breast cancer risk in women aged 40 to 65 years, but there is limited evidence of its association with risk of breast cancer among women aged 65 years or older.

**Objective:**

To compare the association between breast density and risk of invasive breast cancer among women aged 65 to 74 years vs women aged 75 years or older and to evaluate whether the association is modified by body mass index (BMI).

**Design, Setting, and Participants:**

This prospective cohort study used data from the Breast Cancer Surveillance Consortium from January 1, 1996, to December 31, 2012, for US women aged 65 years or older who underwent screening mammography. Data were analyzed from January 1, 2018, to December 31, 2020.

**Exposures:**

Breast Imaging Reporting and Data System breast density category, age, and BMI.

**Main Outcomes and Measures:**

The 5-year cumulative incidence of invasive breast cancer by level of breast density (almost entirely fat, scattered fibroglandular densities, or heterogeneous or extreme density) and age (65-74 vs ≥75 years) was calculated using weighted means. Cox proportional hazards models were fit to estimate the association of breast density with invasive breast cancer risk. The likelihood ratio test was used to test the interaction between BMI and breast density.

**Results:**

A total of 221 714 screening mammograms from 193 787 women were included in the study; a total of 38% of the study population was aged 75 years or older. Of the mammograms, most were from women aged 65 to 74 years (64.6%) and non-Hispanic White individuals (81.4%). The 5-year cumulative incidence of invasive breast cancer increased in association with increasing breast density among women aged 65 to 74 years (almost entirely fatty breasts: 11.3 per 1000 women [95% CI, 10.4-12.5 per 1000 women]; scattered fibroglandular densities: 17.2 per 1000 women [95% CI, 16.1-17.9 per 1000 women]; extremely or heterogeneously dense breasts: 23.7 per 1000 women [95% CI, 22.4-25.3 per 1000 women]) and among those aged 75 years or older (fatty breasts: 13.5 per 1000 women [95% CI, 11.6-15.5]; scattered fibroglandular densities: 18.4 per 1000 women [95% CI, 17.0-19.5 per 1000 women]; extremely or heterogeneously dense breasts: 22.5 per 1000 women [95% CI, 20.2-24.2 per 1000 women]). Extreme or heterogeneous breast density was associated with increased risk of breast cancer compared with scattered fibroglandular breast density in both age categories (65-74 years: hazard ratio [HR], 1.39 [95% CI, 1.28-1.50]; ≥75 years: HR, 1.23 [95% CI, 1.10-1.37]). Women with almost entirely fatty breasts had a decrease of approximately 30% (range, 27%-34%) in the risk of invasive breast cancer compared with women with scattered fibroglandular breast density (65-74 years: HR, 0.66 [95% CI, 0.58-0.75]; ≥75 years: HR, 0.73; 95% CI, 0.62-0.86). Associations between breast density and breast cancer risk were not significantly modified by BMI (for age 65-74 years: likelihood ratio test, 2.67; *df*, 2; *P* = .26; for age ≥75 years, 2.06; *df*, 2; *P* = .36).

**Conclusions and Relevance:**

The findings suggest that breast density is associated with increased risk of invasive breast cancer among women aged 65 years or older. Breast density and life expectancy should be considered together when discussing the potential benefits vs harms of continued screening mammography in this population.

## Key Points

**Question:**

Is breast density associated with invasive breast cancer among women aged 65 years or older, and does body mass index (BMI) modify any associations between breast density and breast cancer risk?

**Findings:**

In this cohort study of 221 714 screening mammograms among 193 787 US women aged 65 years or older, heterogeneous or extreme breast density vs scattered fibroglandular density was associated with an increased risk of invasive breast cancer among women aged 65 to 74 years and among those aged 75 years or older with a BMI of 25 or higher. No interaction between BMI and breast density was found in either age group.

**Meaning:**

The findings suggest that breast density is associated with an increased risk of invasive breast cancer among women aged 65 years or older.

## Teaser

This cohort study used data from the Breast Cancer Surveillance Consortium to examine associations between incidence of invasive breast cancer and breast density, body mass index, and age among women aged 65 years or older.

## Introduction

Breast density, a potential intermediate phenotype in the molecular pathway of breast cancer, is a measure of the amount of radiopaque fibroglandular tissue in the breast that consists of epithelial tissue and stroma as opposed to surrounding fat tissue that appears radiolucent on mammography.^1,2^ Evidence from the US Breast Cancer Surveillance Consortium (BCSC) estimates that approximately 27.6 million women (43.3%) aged 40 to 74 years in the US have dense breasts (heterogeneously or extremely dense), whereas the prevalence of dense breasts is only 28% among women aged 75 years or older.^3,4^ A systematic review^1^ comparing breast density among age groups concluded that breast density decreased in association with increasing age in both the premenopausal and postmenopausal period, with greater decreases observed in the postmenopausal period.

Rapid population aging in the US and worldwide underscores the need to examine factors associated with breast cancer in older women that can be assessed along with life expectancy to inform precision breast cancer screening.^5,6^ Although breast density is a factor associated with breast cancer among women aged 40 to 74 years,^1,7,8,9,10^ the association of breast density with breast cancer among women aged 75 or older is not well established.^10^ Epidemiological evidence suggests an association between breast density and breast cancer among women younger than 65 years^3,8^ and premenopausal women.^11^ The likelihood that a woman will have dense breasts based on the Breast Imaging Reporting and Data System (BI-RADS) decreases in association with increasing body mass index (BMI).^4^ Thus, evaluation of whether BMI modifies any association of breast density with breast cancer among older women is important.^12,13^ Current breast density notification laws in the US may create the expectation that supplemental screening will benefit women with dense breasts, but this has not been established for older women.^14^ Assessing the association of breast density with breast cancer in the context of advancing age and whether any associations are modified by BMI is crucial for understanding the biologic characteristics of breast cancer, estimating the risk of breast cancer later in life, and identifying population subgroups who might benefit from life expectancy–based screening.^15^

We used data from the population-based BCSC cohort to quantify the risk of invasive breast cancer according to BI-RADS breast density categories and compared the association between breast density and risk of invasive breast cancer among women aged 75 years or older vs 65 to 74 years. We also assessed whether associations were modified by BMI. The goal of this study was to identify population subgroups who may be at increased risk of breast cancer, especially because the US Preventive Service Task Force guidelines state that the current evidence is considered insufficient to recommend routine breast cancer screening for women aged 75 years or older.^16,17^

## Methods

### Inclusion and Exclusion Criteria

This cohort study used data from the National Cancer Institute–funded BCSC, a community-based, geographically and racially/ethnically diverse cohort study that broadly represents the population of women who receive screening mammography in the US.^18,19^ The BCSC registries collect information on demographics, risk factors, clinical history, pathologic features, and mammography indication and results.^20^ Breast cancer diagnoses and tumor characteristics are obtained by linking BCSC data to pathology services; regional Surveillance, Epidemiology, and End Results programs; and/or state tumor registries. Data are pooled at a central Statistical Coordinating Center (SCC). Each registry and the SCC received institutional review board approval for active or passive consenting processes or a waiver of consent to enroll participants, link data, and perform analytic studies. All procedures were compliant with the Health Insurance Portability and Accountability Act, and all registries and the SCC have received a Federal Certificate of Confidentiality and other protection for the identities of women, physicians, and facilities who are subjects of this research. This study followed the Strengthening the Reporting of Observational Studies in Epidemiology (STROBE) reporting guideline.

We included 7 BCSC registries located in New Hampshire, North Carolina, the San Francisco Bay Area, western Washington, New Mexico, Colorado, and Vermont. The sample included 221 714 mammograms from 193 787 women aged 65 years or older that were performed between January 1, 1996, and December 31, 2012, and included the BCSC’s comprehensive longitudinal collection of breast density as recorded by interpreting radiologists.^21^ For women with more than 1 mammogram during the study period, we selected the first mammogram performed within each of 2 age subdivisions: 65 to 74 years and 75 years or older. We excluded women with a breast cancer diagnosis before or within 3 months after their first eligible mammogram because we set out to examine incident breast cancer risk. Women were also excluded if they had breast implants or had undergone mastectomy or if their breast density or BMI was not available. Data were analyzed from January 1, 2018, to December 31, 2020.

### Exposure and Outcomes of Interest

Breast density was the study’s primary exposure and was classified by interpreting radiologists as part of their clinical interpretation of mammograms using the 4 American College of Radiology BI-RADS breast density categories^22^: a, almost entirely fat; b, scattered fibroglandular densities; c, heterogeneously dense; and d, extremely dense. We grouped the heterogeneously dense (27.8%) and extremely dense (3.7%) categories together (total, 31.5% of the mammograms) because extremely dense breasts are infrequent in older women.^11^ The main outcome variable was incident invasive breast cancer within 3 months to 10 years after the assessment of breast density.

### Measurement of Covariates

Covariate information was obtained from self-report at the time of mammography and included age, first-degree family history of breast cancer, race/ethnicity, weight, height, current postmenopausal hormone therapy use at the time of mammography, and history of breast biopsy. Self-reported height and weight were used to calculate BMI (calculated as weight in kilograms divided by height in meters squared), which was categorized as less than 18.5, 18.5 to 24.9, 25.0 to 29.9, 30.0 to 34.9, or 35.0 or greater based on World Health Organization criteria.^12^ However, in subgroup analysis, we dichotomized BMI as less than 25 (normal weight) vs 25 or higher (overweight or obese) to avert small sample sizes. Race/ethnicity was grouped as non-Hispanic White, non-Hispanic Black, Asian or Pacific Islander, Hispanic, other (Native Hawaiian or Pacific Islander and American Indian or Alaskan Native), and mixed race/ethnicity. Age at the time of mammography was categorized based on US Preventive Service Task Force screening cutoffs^17^: women aged 65 to 74 years, for whom screening is recommended, and women aged 75 years or older, for whom there is no recommendation for or against screening. Use of hormone therapy at the time of mammography was classified dichotomously based on women’s self-report. Prior diagnoses of benign breast disease were collected by BCSC registries from pathology reports and radiology systems. We grouped all diagnoses from each patient’s pathology reports into 1 category based on the most invasive finding: nonproliferative, proliferative without atypia, proliferative with atypia, or lobular carcinoma in situ.^23,24^ We classified a biopsy as indicating an unknown diagnosis if a woman reported undergoing a prior biopsy but pathology results were not available.

### Statistical Analysis

By design, all women who had missing age and breast density data as well as those with missing values for specific covariates were excluded from all data analyses based on those variables. Using ascertained breast density as the unit of analysis, we evaluated the distribution of breast density by demographic and clinical factors that are associated with breast cancer risk in older women. We used separate models for women aged 75 years or older vs those aged 65 to 74 years. The Fine and Gray method^25^ was used to estimate the 5-year cumulative incidence of invasive breast cancer by breast density, BMI, and age group after accounting for mortality from causes other than breast cancer.^26,27^ The model was adjusted for the patients’ BCSC registry and factors associated with breast cancer risk, including family history of breast cancer, race/ethnicity, hormone therapy use, and benign breast disease (Table 1). Results were based on marginal standardization with the predicted function summed to a weighted mean according to the observed covariate distribution in the study population. We used Cox proportional hazards regression analysis to model the time to diagnosis of invasive breast cancer according to breast density and age group, adjusted for the patient’s BCSC registry and factors associated with breast cancer risk, including family history of breast cancer, race/ethnicity, hormone therapy use, benign breast disease, and BMI (Table 1). The follow-up time started 3 months after the first eligible mammogram. Women were censored at the time of death, diagnosis of ductal carcinoma in situ, mastectomy, end of complete cancer capture, or 10 years after study entry, whichever happened first. To assess whether BMI modified the association of breast density with breast cancer risk among older women, we fitted separate models among women aged 65 to 74 years and those aged 75 years or older to test the interaction between BMI (<25 vs ≥25) and breast density using the likelihood ratio test. Two-sided statistical tests with *P* < .05 were considered statistically significant. Statistical analyses were conducted using R, version 3.4.3 (R Project for Statistical Computing).

**Table 1.** Characteristics of Women in the BCSC by Breast Density on Screening Mammograms

Characteristic	Mammograms, No. (%)^**a**^			
	All (N = 221 714)	Breast density^**b**^		
		Almost entirely fat (n = 37 379)	Scattered fibroglandular densities (n = 114 404)	Heterogeneously or extremely dense (n = 69 931)
Age at study entry, y				
65-74	143 118 (64.6)	23 661 (16.5)	73 507 (51.4)	45 950 (32.1)
≥75	78 596 (35.4)	13 718 (17.5)	40 897 (52.0)	23 981 (30.5)
Race/ethnicity^c^				
White, non-Hispanic	173 347 (81.4)	27 307 (15.8)	90 304 (52.1)	55 736 (32.2)
Black, non-Hispanic	4072 (1.9)	898 (22.1)	2077 (51.0)	1097 (26.9)
Asian or Pacific Islander	13 613 (6.4)	2356 (17.3)	6036 (44.3)	5221 (38.4)
Hispanic	19 272 (9.0)	4380 (22.7)	9984 (51.8)	4908 (25.5)
Other or mixed^d^	2680 (1.3)	495 (18.5)	1386 (51.7)	799 (29.8)
Unknown	8730 (3.9)	1943 (22.3)	4617 (52.9)	2170 (24.9)
Benign breast disease				
None, no prior biopsy	173 837 (78.4)	31 754 (18.3)	90 919 (52.3)	51 164 (29.4)
Prior biopsy, unknown diagnosis	38 924 (17.6)	4522 (11.6)	19 086 (49.0)	15 316 (39.3)
Nonproliferative	5589 (2.5)	764 (13.7)	2793 (50.0)	2032 (36.4)
Proliferative without atypia	2636 (1.2)	284 (10.8)	1281 (48.6)	1071 (40.6)
Proliferative with atypia	560 (0.3)	39 (7.0)	243 (43.4)	278 (49.6)
LCIS	168 (0.1)	16 (9.5)	82 (48.8)	70 (41.7)
Postmenopausal hormone therapy use^c^				
No	158 560 (77.4)	29 690 (18.7)	83 884 (52.9)	44 986 (28.4)
Yes	46 204 (22.6)	5047 (10.9)	21 688 (46.9)	19 469 (42.1)
Unknown	16 950 (7.6)	2642 (15.6)	8832 (52.1)	5476 (32.3)
BMI^c^				
<18.5	4738 (2.1)	367 (7.7)	1762 (37.2)	2609 (55.1)
18.5 to <25	92 171 (41.6)	10 154 (11.0)	44 625 (48.4)	37 392 (40.6)
25 to <30	73 905 (33.3)	13 189 (17.8)	40 437 (54.7)	20 279 (27.4)
30 to <35	34 016 (15.3)	8207 (24.1)	18 779 (55.2)	7030 (20.7)
≥35	16 884 (7.6)	5462 (32.4)	8801 (52.1)	2621 (15.5)
Invasive breast cancer				
No	216 645 (97.7)	36 854 (17.0)	111 812 (51.6)	67 979 (31.4)
Yes	5069 (2.3)	525 (10.4)	2592 (51.1)	1952 (38.5)

Abbreviations: BCSC, Breast Cancer Surveillance Consortium; BI-RADS, Breast Imaging Reporting and Data System; BMI, body mass index (calculated as weight in kilograms divided by height in meters squared); LCIS, lobular carcinoma in situ.

^a^ Some columns do not add to 100% owing to rounding.

^b^ Almost entirely fat was categorized as BI-RADS a; scattered fibroglandular densities, BI-RADS b; and heterogeneously or extremely dense, BI-RADS c and d. Categories c and d were grouped together because they are not prevalent in older women.

^c^ Missing responses were excluded.

^d^ Other or mixed includes Native Hawaiian or Pacific Islander, American Indian or Alaskan Native, and women with mixed race/ethnicity.

## Results

### Characteristics of the Study Population

A total of 221 714 screening mammograms from 193 787 women were included in the study; 38% of the study population was aged 75 years or older. During a mean follow-up of 6.3 years, 5069 invasive breast cancers were diagnosed. Of the included mammograms, most were from women aged 65 to 74 years (64.6%) and 35.4% were from women aged 75 years or older (Table 1). Most mammograms were from non-Hispanic White women (81.4%), followed by Hispanic women (9.0%), women who were Asian or Pacific Islanders (6.4%), and Black, non-Hispanic women (1.9%). With regard to breast density, among women aged 65 to 74 years, 16.5% had BI-RADS a, 51.4% had BI-RADS b, and 32.1% had BI-RADS c or d. Among women aged 75 years or older, 17.5% had BI-RADS a, 52.0% had BI-RADS b, and 30.5% had BI-RADS c or d (Figure). Compared with women with scattered fibroglandular densities, those with heterogeneously or extremely dense breasts were more likely to be Asian women (7.7% vs 5.5%), less likely to have benign breast disease (73.2% vs 79.5%), more likely to have a normal BMI (53.5% vs 39.0%), and more likely to currently use postmenopausal hormone therapy (30.2% vs 20.5%). Characteristics of the study population are shown in eTables 1 and 2 in Supplement 1.

**Figure.**
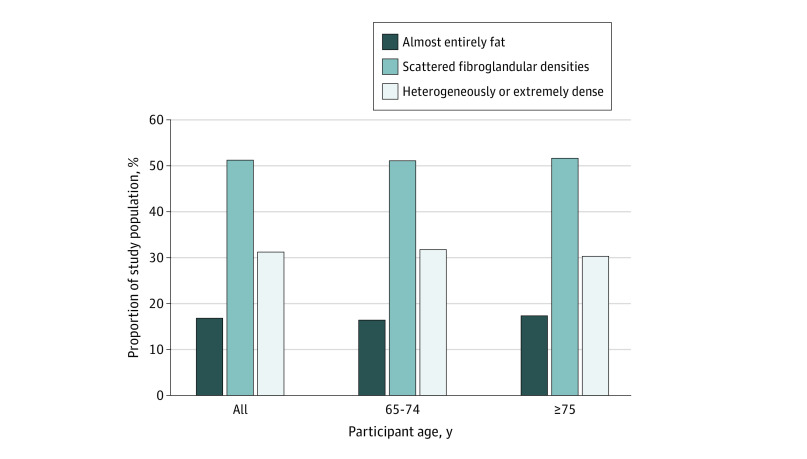
Distribution of Breast Density by Age Group Density categories based on the Breast Imaging Reporting and Data System.

### Five-Year Cumulative Incidence of Invasive Breast Cancer by Breast Density, Age Group, and BMI

In general, we observed higher rates of breast cancer associated with increasing BI-RADS density by BMI categories in both age groups (Table 2). Among women aged 65 to 74 years, the 5-year cumulative incidence of invasive breast cancer increased in association with increasing breast density: 11.3 per 1000 women (95% CI, 10.4-12.5 per 1000 women) in the group whose breasts were almost entirely fat, 17.2 per 1000 women (95% CI, 16.1-17.9 per 1000 women) in the group with scattered fibroglandular density, and 23.7 per 1000 women (95% CI, 22.4-25.3 per 1000 women) in the group with heterogeneously or extremely dense breasts. Among women aged 75 years or older, the cumulative incidence also increased in association with increasing breast density: 13.5 per 1000 women (95% CI, 11.6-15.5 per 1000 women) in the group with fatty breasts, 18.4 per 1000 women (95% CI, 17.0-19.5 per 1000 women) in the group with scattered fibroglandular density, and 22.5 per 1000 women (95% CI, 20.2-24.2 per 1000 women) in the group with heterogeneously or extremely dense breasts.

**Table 2.** Five-Year Cumulative Incidence of Invasive Breast Cancer Among Women in the BCSC by Breast Density, BMI, and Age Group

Variable	Incidence of invasive breast cancer, per 1000 women (95% CI), by breast density^a^		
	Almost entirely fat	Scattered fibroglandular densities	Heterogeneously or extremely dense
Age, y^b^			
65-74	11.3 (10.4-12.5)	17.2 (16.1-17.9)	23.7 (22.4-25.3)
≥75	13.5 (11.6-15.5)	18.4 (17.0-19.5)	22.5 (20.2-24.2)
Age 65-74 y^c^			
BMI <25	8.2 (6.3-13.4)	15.3 (13.5-17.2)	20.6 (18.2-22.9)
BMI ≥25	12.3 (11.3-15.1)	17.3 (16.7-20.6)	23.9 (23.2-29.2)
Age ≥75 y^c^			
BMI <25	13.5 (10.6-16.4)	16.3 (13.9-18.2)	18.3 (15.9-21.4)
BMI ≥25	15.0 (11.9-17.3)	21.3 (18.0-23.5)	27.2 (22.1-30.8)

Abbreviations: BCSC, Breast Cancer Surveillance Consortium; BI-RADS, Breast Imaging Reporting and Data System; BMI, body mass index (calculated as weight in kilograms divided by height in meters squared).

^a^ Almost entirely fat was categorized as BI-RADS a; scattered fibroglandular densities, BI-RADS b; and heterogeneously or extremely dense, BI-RADS c and d. Categories c and d were grouped together because they are not prevalent in older women.

^b^ Multivariable models were adjusted for first-degree family history of breast cancer, the patient’s BCSC registry, race/ethnicity, postmenopausal hormone therapy use, BMI, and benign breast disease.

^c^ Multivariable models were adjusted for first-degree family history of breast cancer, the patient’s BCSC registry, race/ethnicity, postmenopausal hormone therapy use, and benign breast disease.

In the group of women aged 65 to 74 years with overweight or obesity, the 5-year cumulative incidence of invasive breast cancer was 12.3 per 1000 women (95% CI, 11.3-15.1 per 1000 women) among those with fatty breasts and 23.9 per 1000 women (95% CI, 23.2-29.2 per 1000 women) among those with heterogeneously or extremely dense breasts. In the group of women aged 65 to 74 years with a BMI less than 25, the cumulative incidence was 8.2 per 1000 women (95% CI, 6.3-13.4 per 1000 women) among those with fatty breasts and 20.6 per 1000 women (95% CI, 18.2-22.9 per 1000 women) among those with heterogeneously or extremely dense breasts.

In the group of women aged 75 or older with overweight or obesity (BMI ≥25), the 5-year cumulative incidence of invasive breast cancer was 15.0 per 1000 women (95% CI, 11.9-17.3 per 1000 women) among those with fatty breasts and 27.2 per 1000 women (95% CI, 22.1-30.8 per 1000 women) among those with heterogeneously or extremely dense breasts. In the group of women aged 75 years or older with a BMI less than 25, the cumulative incidence was 13.5 per 1000 women (95% CI, 10.6-16.4 per 1000 women) among those with fatty breasts and 18.3 per 1000 women (95% CI, 15.9-21.4 per 1000 women) among those with heterogeneously or extremely dense breasts.

### Association of Breast Density, Age Group, and BMI With Risk of Invasive Breast Cancer

Compared with scattered fibroglandular breast densities, heterogeneous or extreme breast density was associated with an increased risk of breast cancer among women aged 65 to 74 years regardless of BMI (overall: hazard ratio [HR], 1.39 [95% CI, 1.28-1.50]; BMI ≥25: HR, 1.39 [95% CI, 1.25-1.54]; BMI <25: HR, 1.35 [95% CI, 1.20-1.54]) (Table 3). Similarly, among women aged 75 years or older, heterogeneous or extreme breast density was associated with an increased risk of breast cancer regardless of BMI (overall: HR, 1.23 [95% CI, 1.10-1.37]; BMI ≥25: HR, 1.29 [95% CI, 1.10-1.49]; BMI <25: HR, 1.15 [95% CI, 0.98-1.36]) (*P* = .14 for interaction for age × density).

**Table 3.** Cox Proportional Hazards Regression Analysis for the Association of Breast Density With Invasive Breast Cancer by Age Group and BMI Among Women in the BCSC

Variable	Hazard ratio (95% CI), by breast density^a^		
	Almost entirely fat	Scattered fibroglandular densities	Heterogeneously or extremely dense
Age, y^b^			
65-74	0.66 (0.58-0.75)	1 [Reference]	1.39 (1.28-1.50)
≥75	0.73 (0.62-0.86)	1 [Reference]	1.23 (1.10-1.37)
Age 65-74 y^c^			
BMI <25	0.53 (0.40-0.72)	1 [Reference]	1.35 (1.20-1.54)
BMI ≥25	0.71 (0.61-0.81)	1 [Reference]	1.39 (1.25-1.54)
Age ≥75 y^c^			
BMI <25	0.82 (0.62-1.09)	1 [Reference]	1.15 (0.98-1.36)
BMI ≥25	0.70 (0.57-0.86)	1 [Reference]	1.29 (1.10-1.49)

Abbreviations: BCSC, Breast Cancer Surveillance Consortium; BI-RADS, Breast Imaging Reporting and Data System; BMI, body mass index (calculated as weight in kilograms divided by height in meters squared).

^a^ Almost entirely fat was categorized as BI-RADS a; scattered fibroglandular densities, BI-RADS b; and heterogeneously or extremely dense, BI-RADS c and d. Categories c and d were grouped together because they are not prevalent in older women.

^b^ Multivariable models were adjusted for first-degree family history, the patient’s BCSC registry, race/ethnicity, BMI, postmenopausal hormone therapy use, and benign breast disease.

^c^ Multivariable models were adjusted for first-degree family history, the patient’s BCSC registry, race/ethnicity, postmenopausal hormone therapy use, and benign breast disease.

Among women aged 65 to 74 years, those with almost entirely fatty breasts had a decreased risk of invasive breast cancer compared with women with scattered fibroglandular breast densities regardless of BMI (overall: HR, 0.66 [95% CI, 0.58-0.75]; BMI ≥25: HR, 0.71 [95% CI, 0.61-0.81]; BMI <25: HR, 0.53 [95% CI, 0.40-0.72]). Similar associations were observed in the group of women aged 75 years or older overall (HR, 0.73; 95% CI, 0.62-0.86) and among those with a BMI of 25 or higher (HR, 0.70; 95% CI, 0.57-0.86), but there was no association among those with a BMI less than 25 (HR, 0.82; 95% CI, 0.62-1.09). There was no significant interaction between BMI and breast density among women aged 65 to 74 years (likelihood ratio test, 2.67; *df*, 2; *P* = .26) and those 75 years or older (likelihood ratio test, 2.06; *df*, 2; *P* = .36).

## Discussion

In this cohort study of the association between BI-RADS breast density categories and the risk of invasive breast cancer by BMI level and age among women aged 65 years or older, we found that breast density was associated with increased breast cancer risk among women aged 65 to 74 years regardless of BMI and among those aged 75 or older with a BMI of 25 or higher. The findings are consistent with those reported in a previous study^3^ of women aged 40 to 74 years that revealed associations between age and most of the breast cancer risk factors investigated, with the association between age and BI-RADS breast density declining as age increased. Although breast density is important in risk assessment and could be evaluated in older women, some risk prediction models do not provide risk estimates for women aged 75 or older.^28,29^ Given that greater breast density as categorized by the BI-RADS remains a factor associated with breast cancer even in older women,^2,3^ information about breast density together with life expectancy may benefit clinical decision-making regarding whether screening after 75 years of age should be continued.

In March 2019, the US Food and Drug Administration recommended changes to the Mammography Quality Standards Act to make it mandatory to report breast density information to both patients and their physicians.^30^ However, how women and their physicians should use this information to inform screening recommendations is unclear, particularly for older women. This study adds to the literature by showing that despite a decrease in breast density associated with increasing age, density continued to be associated with a modest increase in breast cancer risk among women aged 65 to 74 years and those aged 75 years or older. This study also showed that the association between BI-RADS breast density categories and breast cancer was statistically significant after adjustment for BMI and other factors. Additional research is needed to elucidate the mechanisms underlying the observed associations between breast density and risk of breast cancer.^31,32^ For example, a potential mechanism is hypercellularity and increased breast epithelium in dense breasts that may lead to increased rates of somatic mutations.^33^ In addition, dense breasts contain a large amount of stroma with aromatase activity, which has been associated with an increased release of estrogen that may lead to carcinogenesis.^34^ Further studies are needed to elucidate these mechanisms among older women.

Although this study showed a significant association between breast density and increased breast cancer risk among older women, the estimates in our study were lower than the estimates reported in a meta-analysis by McCormack and dos Santos Silva^35^ based on quantitative measures of density percentages. In 2 studies,^36,37^ the pooled relative risk of breast cancer was increased in women with greater breast density (ie, pooled odds ratio for heterogeneously dense breasts, 2.81 [95% CI, 2.13-3.71]; odds ratio for extremely dense breasts, 4.08 [95% CI, 2.96-5.63]).^35^ Although Lam et al^37^ found that the risk of breast cancer increased in association with increased breast density and vice versa after adjusting for a patient’s BMI, the study included a small group of women aged 75 years or older, and the estimates did not take into account factors associated with breast cancer. Similarly, in a study of 61 844 women, of whom 4137 (6.7%) were aged 75 years or older, Vacek et al^36^ observed an increased breast cancer risk in association with increasing breast density but did not stratify results based on age. We extended the results from these studies by quantifying the association between BI-RADS breast density categories and breast cancer risk in a population-based cohort of older women who underwent screening mammography in US community practice.

As newer and more advanced breast density assessment techniques are developed,^38^ evaluation of the diffusion of such innovations among older women with an aim of developing individualized screening strategies will be important, particularly among women aged 75 years or older, for whom guidelines regarding screening mammography remain unclear. With the aging of the population in the US and worldwide, it is becoming increasingly important to implement life expectancy–based screening strategies^15,39^ that consider breast cancer risk so that older women who are likely to benefit can continue to undergo screening and risk of harms such as overdiagnosis and overtreatment can be reduced among those unlikely to benefit.

### Strengths and Limitations

This study has strengths. We used, to our knowledge, the largest population-based sample of socioeconomically diverse older women undergoing screening mammography in US community practice. In addition, the study was conducted with prospective cohort design and included complete breast cancer follow-up. We were also able to account for the several confounding factors, including family history of breast cancer, race/ethnicity, postmenopausal hormone therapy use, BMI, and benign breast disease. Furthermore, we examined the association of breast density with breast cancer separately by age group, which included a large proportion of women aged 75 years or older.

This study also has limitations. It relied on BI-RADS breast density measurements reported by interpretive radiologists as part of clinical practice at multiple radiology facilities.^40,41^ Of note, interobserver agreement on the BI-RADS categories is moderate.^42^ Although the lack of quantitative measures of breast density in this study may be considered a limitation, a strength of the BI-RADS measurement categories is their clinical relevance; the categorical assessment of BI-RADS density is the measure provided clinically on all mammograms performed in the US. The BI-RADS measurements have evolved over time, with the 2013 (5th) edition being the most recent. Although this study’s population included women from 1996 to 2012, we used the latest BI-RADS measurements to evaluate the association of breast density with breast cancer risk. As the methods for measuring breast density evolve, future studies should continue to evaluate associations between breast cancer risk and breast density, especially among older women. Another limitation of this study was the use of self-reported height and weight; a considerable amount of these data was missing, mostly because some BCSC facilities did not collect this information during some or all years.

## Conclusions

In this cohort study, we observed an association between increased density as categorized by the BI-RADS and an increased risk of invasive breast cancer, but low breast density (ie, fatty breasts) was associated with a decreased risk of invasive breast cancer across age groups. The positive associations found in this study between breast density and breast cancer among women aged 75 years or older suggest that breast density and life expectancy should be considered together when discussing the potential benefits and harms of continued screening mammography in this population.^43^

## Notes

Supplement 1. **eTable 1.** Characteristics of Women Aged 65-74 Years **eTable 2.** Characteristics of Women Aged 75 Years or Older

Supplement 2.
The Breast Cancer Surveillance Consortium
