# Supplementary material for: Association of Breast Density With Breast Cancer Risk Among Women Aged 65 Years or Older by Age Group and Body Mass Index
Source: JAMA Netw Open. 2021 Aug 26;4(8):e2122810. doi: 10.1001/jamanetworkopen.2021.22810 (PMC8391100; doi:10.1001/jamanetworkopen.2021.22810)
Supplement: Supplement 1. — eTable 1. Characteristics of Women Aged 65-74 Years eTable 2. Characteristics of Women Aged 75 Years or Older [file jamanetwopen-e2122810-s001.pdf]

Supplementary Online Content

Advani SM, Zhu W, Demb J, et al; Breast Cancer Surveillance Consortium. Association of breast density with breast cancer risk among women aged 65 years or older by age group and body mass index. *JAMA Netw Open*. 2021;4(8):e2122810. doi:10.1001/jamanetworkopen.2021.22810

eTable 1. Characteristics of Women Aged 65-74 Years
eTable 2. Characteristics of Women Aged 75 Years or Older

This supplementary material has been provided by the authors to give readers additional information about their work.

© 2021 Advani SM et al. *JAMA Network Open*.

eTable 1: Characteristics of women aged 65-74 years*

	All mammograms (N=143,118)	Almost entirely fat BI-RADS a (N=23,661)	Scattered fibroglandular densities BI-RADS b (N=73,507)	Heterogeneously/ extremely dense BI-RADS c and d (N=45,950)
Race/ethnicity (N, % among non-missings)				
White, non-Hispanic	110258 (80)	17196 (76.5)	57163 (80.8)	35899 (80.5)
Black, non-Hispanic	2715 (2)	593 (2.6)	1378 (1.9)	744 (1.7)
Asian/Pacific Islander	9327 (6.8)	1458 (6.5)	4149 (5.9)	3720 (8.3)
Hispanic	13528 (9.8)	2886 (12.8)	7025 (9.9)	3617 (8.1)
Other or mixed	1948 (1.4)	348 (1.5)	1011 (1.4)	589 (1.3)
Unknown, %	5342 (3.7)	1180 (5)	2781 (3.8)	1381 (3)
Benign breast disease (N, %)				
None (no prior biopsy)	111989 (78.2)	20057 (84.8)	58403 (79.5)	33529 (73)
Prior biopsy, unknown diagnosis	25086 (17.5)	2896 (12.2)	12153 (16.5)	10037 (21.8)
Non-proliferative	3799 (2.7)	504 (2.1)	1878 (2.6)	1417 (3.1)
Proliferative without atypia	1742 (1.2)	172 (0.7)	860 (1.2)	710 (1.5)
Proliferative with atypia	394 (0.3)	25 (0.1)	163 (0.2)	206 (0.4)
LCIS	108 (0.1)	7 (0)	50 (0.1)	51 (0.1)
Postmenopausal hormone therapy use (N, % among non-missings)				
No	99047 (74.8)	18449 (83.5)	52192 (76.7)	28406 (67)
Yes	33434 (25.2)	3639 (16.5)	15816 (23.3)	13979 (33)
Unknown, %	10637 (7.4)	1573 (6.6)	5499 (7.5)	3565 (7.8)
Body mass index kg/m^2 (N, % among non-missings)				
<18.5	2461 (1.7)	171 (0.7)	894 (1.2)	1396 (3)
[18.5,25)	56655 (39.6)	5623 (23.8)	27006 (36.7)	24026 (52.3)
[25,30)	47802 (33.4)	8164 (34.5)	26027 (35.4)	13611 (29.6)
[30,35)	23306 (16.3)	5516 (23.3)	12864 (17.5)	4926 (10.7)
⩾35	12894 (9)	4187 (17.7)	6716 (9.1)	1991 (4.3)
Invasive Breast Cancer				
No	139792 (97.7)	23337 (98.6)	71825 (97.7)	44630 (97.1)
Yes	3326 (2.3)	324 (1.4)	1682 (2.3)	1320 (2.9)

© 2021 Advani SM et al. *JAMA Network Open*.

eTable 2: Characteristics of women aged 75 years or older*

	All mammograms	Almost entirely fat	Scattered fibroglandular densities	Heterogeneously/
	(N=78,596)	BI-RADS a	BI-RADS b	extremely dense
		(N=13,718)	(N=40,897)	BI-RADS c and d
				(N=23,981)
Race/ethnicity (N, % among non-missings)				
White, non-Hispanic	63089 (83.9)	10111 (78)	33141 (84.8)	19837 (85.5)
Black, non-Hispanic	1357 (1.8)	305 (2.4)	699 (1.8)	353 (1.5)
Asian/Pacific Islander	4286 (5.7)	898 (6.9)	1887 (4.8)	1501 (6.5)
Hispanic	5744 (7.6)	1494 (11.5)	2959 (7.6)	1291 (5.6)
Other or mixed	732 (1)	147 (1.1)	375 (1)	210 (0.9)
Unknown, %	3388 (4.3)	763 (5.6)	1836 (4.5)	789 (3.3)
Benign breast disease (N, %)				
None (no prior biopsy)	61848 (78.7)	11697 (85.3)	32516 (79.5)	17635 (73.5)
Prior biopsy, unknown diagnosis	13838 (17.6)	1626 (11.9)	6933 (17)	5279 (22)
Non-proliferative	1790 (2.3)	260 (1.9)	915 (2.2)	615 (2.6)
Proliferative without atypia	894 (1.1)	112 (0.8)	421 (1)	361 (1.5)
Proliferative with atypia	166 (0.2)	14 (0.1)	80 (0.2)	72 (0.3)
LCIS	60 (0.1)	9 (0.1)	32 (0.1)	19 (0.1)
Postmenopausal hormone therapy use (N, % among non-missings)				
No	59513 (82.3)	11241 (88.9)	31692 (84.4)	16580 (75.1)
Yes	12770 (17.7)	1408 (11.1)	5872 (15.6)	5490 (24.9)
Unknown, %	6313 (8)	1069 (7.8)	3333 (8.1)	1911 (8)
Body mass index kg/m^2 (N, % among non-missings)				
<18.5	2277 (2.9)	196 (1.4)	868 (2.1)	1213 (5.1)
[18.5,25)	35516 (45.2)	4531 (33)	17619 (43.1)	13366 (55.7)
[25,30)	26103 (33.2)	5025 (36.6)	14410 (35.2)	6668 (27.8)
[30,35)	10710 (13.6)	2691 (19.6)	5915 (14.5)	2104 (8.8)

© 2021 Advani SM et al. *JAMA Network Open*.

⩾35	3990 (5.1)	1275 (9.3)	2085 (5.1)	630 (2.6)
Invasive Breast Cancer				
No	76853 (97.8)	13517 (98.5)	39987 (97.8)	23349 (97.4)
Yes	1743 (2.2)	201 (1.5)	910 (2.2)	632 (2.6)

© 2021 Advani SM et al. *JAMA Network Open*.
