# Supplementary material for: Association of Breast Density With Breast Cancer Risk Among Women Aged 65 Years or Older by Age Group and Body Mass Index
Source: JAMA Netw Open. 2021 Aug 26;4(8):e2122810. doi: 10.1001/jamanetworkopen.2021.22810 (PMC8391100; doi:10.1001/jamanetworkopen.2021.22810)
Supplement: Supplement 2. — The Breast Cancer Surveillance Consortium [file jamanetwopen-e2122810-s002.pdf]

Supplemental Online Content: Nonauthor Collaborators

*Indicates required information. Only first name, last name, and suffix will appear in PubMed.

*Group Name(s): Breast Cancer Surveillance Consortium							
***First Name and Middle Initial(s)**	***Last Name**	***Suffix (eg, Jr, III)**	**Academic Degrees**	**Institution**	**Location (city, state/province, country)**	**Role or Contribution, eg, chair, principal investigator**	**Group (if more than 1 Group listed in the byline) and/or Subgroup (eg, Steering Committee)**
Brian L	Sprague		PhD	Department of Surgery, University of Vermont Larner College of Medicine, Burlington, VT	Burlington, VA, USA	Steering committee member	Breast Cancer Survelience Consortium
Tracy	Onega		PhD	Department of Population Sciences, University of Utah, Salt Lake City, UT	Salt Lake City, Utah, USA	Steering committee member	Breast Cancer Survelience Consortium
Louise M	Henderson		PhD	Department of Radiology, University of North Carolina at Chapel Hill, NC	Chapel Hill, NC	Steering committee member	Breast Cancer Survelience Consortium
Diana SM	Buist		PhD	Kaiser Permanente Washington Health Research Institute, Seattle, WA	Seattle, WA	Steering committee member	Breast Cancer Survelience Consortium
Karla	Kerlikowske		MD	Department of Medicine, University of California, San Francisco, San Francisco, CA	San Francisco, CA	Steering committee member	Breast Cancer Survelience Consortium
Diana L	Miglioretti		MD	Department of Public Health Sciences, School of Medicine, University of California, Davis, Davis, CA	Davis, CA	Steering committee member	Breast Cancer Survelience Consortium
WeiWei	Zhu		MS	Department of Public Health Sciences, School of Medicine, University of California	Salt Lake City, Utah, USA	Co-I	Breast Cancer Surveillance Consortium
